# The role of serum adipokines in predicting response to neoadjuvant therapy in patients with locally advanced rectal cancer

**DOI:** 10.1590/1806-9282.20251705

**Published:** 2026-08-07

**Authors:** Husnu Ozan Sevik, Omer Akay, Mert Guler, Anil Demir, Soykan Arikan, Ufuk Oguz Idiz, Mert Mahsuni Sevinc, Aziz Ari, Abdullah Sakin, Cihad Tatar

**Affiliations:** 1 University of Health Sciences, Istanbul Training and Research Hospital, Department of General Surgery – Istanbul, Turkey.; 2 University of Health Sciences, Basaksehir Cam and Sakura City Hospital, Department of General Surgery, Division of Surgical Oncology – Istanbul, Turkey.; 3 Bahçelievler Medipol Hospital, Department of Internal Medicine, Division of Medical Oncology – Istanbul, Turkey.; 4 Acibadem Taksim Hospital, Department of General Surgery – Istanbul, Turkey.

**Keywords:** Rectal cancer, Adipokines, Adiponectin, Leptin, Resistin

## Abstract

**OBJECTIVE:**

The response to neoadjuvant therapy in patients with locally advanced rectal cancer varies considerably among individuals. While some patients achieve a complete pathological response, others may even experience disease progression during treatment. The aim of the study was to investigate the predictive value of serum adipokines in determining the response to neoadjuvant therapy.

**METHODS:**

This prospective study included patients diagnosed with rectal cancer and scheduled for neoadjuvant therapy at our institution between September 2020 and September 2022. Before the treatment, blood samples were collected from the patients, and levels of serum adipokines (adiponectin, leptin, resistin, and adipsin) were measured. Following surgical resection, the Modified Ryan Scoring System was used to assess the response to neoadjuvant chemoradiotherapy. Patients were then divided into two groups according to the response to the treatment (Group 1: tumor regression grade 0; Group 2: tumor regression grade 1–3).

**RESULTS:**

In the 58 patients included, no significant differences were observed in terms of demographics, tumor characteristics, or serum biochemical parameters. However, levels of adiponectin (p=0.039) and resistin (p=0.017) were significantly lower in the pathologic complete response group.

**CONCLUSION:**

Adiponectin and resistin might contribute to anticipating response to neoadjuvant chemoradiotherapy in patients with locally advanced rectal cancer. The incorporation of these markers into personalized therapy approaches has the potential to improve correct decision-making in the management of locally advanced rectal cancer.

## INTRODUCTION

Neoadjuvant chemoradiotherapy (nCRT) followed by surgery is a widely accepted approach in the treatment of patients with locally advanced rectal cancer (LARC). A pathologic complete response (pCR) to nCRT is strongly associated with favorable oncologic outcomes, including improved overall survival (OS) and disease-free survival (DFS)^1^. Nevertheless, pCR is achieved in only 20–22% of patients^2,3^, while up to 40% exhibit resistance to nCRT, characterized by disease progression or minimal regression to stable disease^4,5^. Given the potential toxicities of nCRT, tailoring the intensity of neoadjuvant therapy according to the anticipated benefit for each patient is crucial^6,7^. Consequently, predicting which patients are likely to achieve pCR has become increasingly important. Although several studies have reported that total neoadjuvant therapy (TNT)—consisting of both chemoradiotherapy and chemotherapy delivered preoperatively—can improve pCR rates^3,8^, reliable predictors of treatment response remain elusive^9^.

Adiponectin, leptin, resistin, and adipsin are serum adipokines synthesized by adipocytes^10^. Through the adipokines they secrete, adipocytes actively interact with cancer cells across multiple organs as integral components of the tumor microenvironment. This interplay has drawn significant attention in cancer research, both for its role in tumorigenesis and its potential as a therapeutic target. Adipokines have been shown to contribute to tumor cell proliferation, migration, invasion, and metastasis^10,11^. Moreover, obesity-related adipokines are closely linked to the incidence of colorectal cancer^12^, and altered adipokine levels have been associated with an increased risk of breast, colorectal, liver, and pancreatic malignancies^10^.

Identifying patients unlikely to respond to nCRT before treatment initiation could allow for upfront surgical resection, thereby reducing the risk of disease progression and distant metastasis during the waiting period between diagnosis and surgery. Conversely, recognizing patients with a high probability of achieving pCR could optimize the selection and duration of neoadjuvant therapy. Therefore, this study aimed to investigate whether serum adipokines possess predictive value in assessing the response to nCRT in patients with LARC.

## METHODS

This prospective study, approved by the Local Human Clinical Studies Ethics Committee [Istanbul Training and Research Hospital (approval number: 2506, dated September 4, 2020)], included patients aged ?18 years with stage II–III rectal cancer who were treated at our tertiary referral hospital between September 1, 2020, and May 1, 2022. Written informed consent was obtained from all participants. The study was registered in a public clinical trial registry (NCT04598984). Exclusion criteria were: impaired pulmonary, hepatic, or renal function; a history of other malignancies within the past 5 years; inability to obtain blood samples prior to neoadjuvant therapy; and failure to undergo surgery at our center following neoadjuvant therapy.

All patients underwent abdominal magnetic resonance imaging (MRI), pelvic MRI, and thoracic computed tomography (CT) for initial staging. Prior to treatment, fasting blood samples (12 h) were collected, centrifuged at 1,000g for 10 min, and serum was separated. Serum aliquots were transferred into Eppendorf tubes and stored at -80°C until analysis. After completion of patient recruitment, samples were thawed and serum adiponectin, leptin, resistin, and adipsin levels were quantified using a flow cytometer (Cube 8^TM^, Sysmex, Japan; cat. no: CY-S-3068R_V3) with a commercial adipokine assay kit (LEGENDplex^TM^ Human Metabolic Panel 1, BioLegend, USA; cat. no: 740212).

All patients received long-course nCRT, consisting of conventional fractionated radiotherapy (1.8–2.0 Gy for 5–6 weeks) combined with concurrent continuous infusion of 5-fluorouracil. Restaging with MRI and endoscopy was performed 8–10 weeks after completion of nCRT, and treatment response was assessed. Patients showing no evidence of distant metastasis or unresectable local disease on restaging, and deemed suitable for surgery by the multidisciplinary tumor board, subsequently underwent total mesorectal excision (TME).

Pathological specimens were evaluated by a single pathologist blinded to clinical data. Pathologic response to nCRT was graded using the modified Ryan scoring system. Patients were classified into two groups according to tumor regression grade (TRG): Group 1 (modified Ryan score 0, complete response) and Group 2 (modified Ryan score 1–3, partial/no response). Demographic characteristics, comorbidities, blood test results, and pathology findings were compared between groups.

### Statistical analysis

Statistical analyses were performed using Statistical Package for the Social Sciences software version 26.0 (IBM Corp., Armonk, NY, USA). The distribution characteristics of continuous variables were examined, and the assumption of normality was assessed using the Shapiro-Wilk test. Data with an approximately normal distribution were presented as mean±standard deviation (SD), whereas non-normally distributed data were expressed as median (interquartile range, IQR). Descriptive statistics were summarized as numbers and percentages for categorical variables. Comparisons of continuous variables between groups were conducted using the Student’s t-test for normally distributed data and the Mann-Whitney U test for non-normally distributed data. Categorical variables were compared using the Chi-square test. A p<0.05 was considered statistically significant.

## RESULTS

A total of 58 patients were included in the study, with a mean age of 59.50±11.92 years and a male-to-female ratio of 31:21. Six patients died due to comorbidities in the preoperative period following nCRT. In addition, seven patients declined surgery after nCRT, and five patients were managed with a watch-and- wait strategy and therefore excluded from the analysis.

Baseline demographic characteristics of the patients, stratified by groups, are presented in Table 1. No significant differences were observed between the groups in this regard. MRI and pathological features of the tumors are summarized in Table 2. The only significant difference was noted in TME quality, with Group 2 showing a significantly higher rate of incomplete TME compared with Group 1 (p=0.019). All patients demonstrated positive nodal involvement, corresponding to Stage III disease.

**Table 1:** Examination of the demographic data of patients with rectal cancer in two groups.

		Group 1 (TRG 0) (n=13)	Group 2 (TRG 1-2-3) (n=45)	p
Age (mean±SD)		58.02±10.04	59.92±12.48	0.629
BMI (kg/m^2^) (mean±SD)		25.61±5.40	25.48±3.23	0.937
Sex n (%)	Female	7 (53.8)	20 (44.4)	0.549
	Male	6 (46.2)	25 (55.6)	
Tobacco n (%)	Don’t use	9 (69.2)	28 (62.2)	0.839
	Ex smoker	3 (23.1)	11 (24.4)	
	Smoker	1 (7.7)	6 (13.3)	
ASA n (%)	1–2	13 (100.0)	41 (91.1)	0.565
	3–4	0 (0.00)	4 (8.9)	
Comorbidity n (%)	Yes	6 (46.2)	13 (28.9)	0.243
	No	7 (53.8)	32 (71.1)	

TRG: tumor regression grade; BMI: body mass index; SD: standard deviation; ASA: American Society of Anesthesiology physical status.

**Table 2:** Clinical features of the tumor in pre-treatment staging and postoperative specimen quality.

		Group 1 (TRG 0) (n=13)	Group 2 (TRG 1-2-3) (n=45)	p
Tumor distance from anal verge (cm) (mean±SD)		8.00±3.05 cm	7.37±3.20 cm	0.536
Tumor localization n (%)	Proximal	6 (46.2)	13 (28.9)	0.363
	Middle	5 (38.5)	17 (37.8)	
	Distal	2 (15.4)	15 (33.3)	
T stage n (%)	c T2	0 (0.0)	1 (2.2)	0.852
	c T3	11 (84.6)	38 (84.4)	
	c T4	2 (15.4)	6 (13.3)	
N stage n (%)	c N1	3 (23.1)	10 (22.2)	0.948
	c N2	10 (76.9)	35 (77.8)	
TME quality n (%)	Incomplete	2 (15.4)	23 (51.1)	**0.019**
	Nearly complete	10 (76.9)	15 (33.3)	
	Complete	1 (7.7)	7 (15.6)	
Removed lymph node (mean±SD)		12.30±2.62	14.06±6.41	0.170
Metastatic lymph node, median (Q1–Q3)		0 (0–1)	1 (0–3)	0.061

TRG: tumor regression grade. TME: total mesorectal excision. SD: standard deviation. IQR: interquartile range. Data with p<0.05 are statistically significant and were therefore highlighted in bold.

Pre-nCRT serum parameters according to groups are shown in Table 3. Significant differences were identified in adiponectin and resistin levels between groups. The mean serum adiponectin level was 28,455.69±4,302.72 ng/mL in Group 1, compared with 69,494.94±10,190.14 ng/mL in Group 2 (p=0.039). Similarly, the mean serum resistin level was 0.99±1.21 ng/mL in Group 1 and 3.96±7.74 ng/mL in Group 2 (p=0.017).

**Table 3:** Examination of blood parameters and serum adipokines of patients with rectal cancer in two groups to assess the response level to neoadjuvant treatment.

	Group 1 (TRG 0) (n=13)	Group 2 (TRG 1-2-3) (n=45)	p
Hemoglobin (g/dL) (mean±SD)	11.65±2.09	16.14±2.29	0.487
Neutrophil (109/L) (mean±SD)	4.70±1.99	3.47±2.13	0.067
Lymphocyte (109/L) (mean±SD)	1.44±1.15	1.31±1.01	0.680
Platelet (109/L) (mean±SD)	271.61±69.60	233.71±76.75	0.115
Albumin (g/dL) (mean±SD)	1.88±1.98	2.53±1.95	0.292
Urea (mg/dL) (mean±SD)	24.92±14.36	16.68±15.68	0.095
Creatinine (mg/dL) (mean±SD)	3.30±3.34	5.40±4.43	0.076
CEA (µg/L) (mean±SD)	21.61±24.16	28.25±20.84	0.378
CA 19-9 (kU/L) (mean±SD)	38.14±29.51	31.65±29.99	0.531
Adiponectin (ng/mL) (mean±SD)	28,455.69±4,302.72	69,494.94±10,190.14	**0.039**
Leptin (ng/mL) (mean±SD)	4.34±7.75	1.98±3.84	0.137
Resistin (ng/mL) (mean±SD)	0.99±1.21	3.96±7.74	**0.017**
Adipsin (ng/mL) (mean±SD)	1,296.82±1,244.77	1,964.11±2,473.06	0.354

TRG: tumor regression grade; SD: standard deviation. CEA: carcinoembryonic antigen; CA: carbohydrate antigen. Data with p<0.05 are statistically significant and were therefore highlighted in bold.

## DISCUSSION

As organ-preserving strategies gain increasing importance in rectal cancer management, the ability to predict pCR following neoadjuvant therapy has become equally critical. Numerous studies have investigated histological, endoscopic, radiological, and biochemical parameters to predict treatment response in patients with LARC; however, no reliable biomarker has yet been established. The interpretation of outcomes is further challenged by patient heterogeneity, tumor-related factors, variations in nCRT protocols, and the lack of a standardized interval between treatment completion and surgery. To minimize these confounders, we employed a uniform nCRT regimen and adopted a standardized 8–10-week interval before surgery. Within this framework, our study demonstrated that serum adiponectin and resistin levels were significantly lower at baseline in patients who subsequently achieved a complete response to nCRT.

Several biomarkers have been proposed in the literature. Wallin et al.^13^ reported that carcinoembryonic antigen (CEA) may serve as a predictive marker for pCR, particularly in non-smokers. Other studies have highlighted the prognostic significance of serum creatinine across various malignancies, including associations with recurrence risk in colorectal cancer^14,15,16,17^. However, in our study, no significant intergroup differences were observed in either CEA or creatinine levels.

Comorbidities and American Society of Anesthesiology physical status (ASA) score have been hypothesized to influence treatment response, but previous studies have found no such association^18^. Conversely, obesity and adipose tissue distribution may affect radiation dose delivery and efficacy^19,20^. Sun et al.^21^, in a retrospective analysis of 522 patients, reported significantly lower tumor regression and pCR rates in patients with BMI (body mass index) >30 kg/m^2^. In our study, however, no significant associations were observed between BMI, ASA score, comorbidities, and pCR.

Tumor size, clinical stage, and tumor distance from the anal verge have also been investigated as potential predictors^6,9,22^. Some studies have suggested that smaller tumor size (<3 cm) independently predicts higher pCR rates^23,24^, though others have reported only marginal differences of 0.5–1 cm between responders and non-responders^13,25,26^. Restivo et al.^27^suggested that tumors located more than 5 cm from the anal verge were associated with higher pCR rates, whereas Patel et al.^28^ reported reduced pCR in tumors located <4 or >8 cm from the anal verge. In our cohort, tumor size and distance from the anal verge, measured uniformly by MRI, showed no significant relationship with TRG.

Adipokines have recently attracted attention for their role in tumor biology. Obesity-related adipokines are closely linked to colorectal cancer incidence^29^, and experimental studies have demonstrated their influence on tumor progression^30^. Elevated resistin levels have been correlated with obesity, increased colorectal cancer risk, liver metastasis, and poorer survival outcomes^31,32^. Leptin expression has been shown to be significantly upregulated in colorectal adenomas and adenocarcinomas compared with normal mucosa^33^. Adiponectin, in contrast, has been studied as a potential protective factor, with reports linking its serum concentrations to disease status and recurrence risk in colorectal cancer^34,35^. However, conflicting results exist regarding its anticancer role^35^.

In our study, both adiponectin and resistin levels were significantly lower in the pCR group. Adiponectin is generally recognized as an anti-inflammatory, anti-diabetic, and anti-atherosclerotic adipokine that suppresses tumor cell proliferation, migration, invasion, and metastasis^10,36^. Conversely, resistin is a pro-inflammatory mediator associated with obesity, insulin resistance, and atherosclerosis, and has been shown to promote tumorigenesis^10,36^. The observation that both adiponectin and resistin were reduced in responders is intriguing, given their opposing biological functions, and suggests that tumor regression mechanisms are likely more complex than can be explained by isolated adipokine measurements. Our findings highlight the need for further research into the role of adipokines in rectal cancer biology and treatment response.

### Limitations

The main limitation of our study is the relatively small sample size, which may have affected the statistical power of the analyses. Larger, multicenter studies are required to validate our findings. Additionally, our cohort did not include patients treated with TNT, which has recently gained widespread adoption. Serum adipokine dynamics in TNT-treated patients may differ and warrant further investigation.

## CONCLUSION

Neoadjuvant therapy is a cornerstone in the management of LARC, with pCR serving as a critical determinant of prognosis and treatment strategy. However, a subset of patients may experience disease progression or distant metastasis despite undergoing nCRT. Reliable biomarkers to predict treatment response remain an unmet need. Our findings suggest that serum adiponectin and resistin may hold potential as predictive markers for treatment response in rectal cancer. These results underscore the importance of further studies on adipokines to support the development of personalized, biomarker-driven strategies for rectal cancer management.

## Data Availability

The datasets generated and/or analyzed during the current study are available from the corresponding author upon reasonable request.
